# Syndrome Differentiation in Chinese Herbal Medicine for Irritable Bowel Syndrome: A Literature Review of Randomized Trials

**DOI:** 10.1155/2013/232147

**Published:** 2013-03-11

**Authors:** Qing Li, Guo-Yan Yang, Jian-Ping Liu

**Affiliations:** Center for Evidence-Based Chinese Medicine, Beijing University of Chinese Medicine, Beijing 100029, China

## Abstract

Traditional Chinese medicine (TCM) has been commonly used for irritable bowel syndrome (IBS). Syndrome differentiation is one of the important characteristics of TCM. To assess the application and basic characteristics of syndrome differentiation in randomized controlled trials (RCTs) of Chinese herbal medicine for IBS, we performed this paper. We conducted electronic searches in main Chinese and English databases till March 2012. A total of 735 RCTs involving 67,784 IBS participants were included. 224 (30.5%) studies applied syndrome differentiation. The major syndromes of IBS patients were the syndrome of liver stagnation and spleen deficiency (56.8%), spleen-stomach weakness (49.4%), spleen-kidney yang deficiency (48.1%), and cold and heat in complexity (29.6%). Herbal formulas were prescribed based on syndrome differentiation in 202 studies. Chinese patent medicine was more commonly used in studies that only enrolled patients with a specific syndrome. 15 studies compared the therapeutic effect among different syndromes, of which 6 studies showed that there were significant differences among different syndromes. The low use of TCM syndrome differentiation in randomized trials of Chinese herbal medicine for IBS results in the poor pertinence of treatment. TCM syndrome differentiation should be used in further studies at the stage of recruitment, treatment, and data analyses.

## 1. Introduction

Irritable bowel syndrome (IBS) is a common functional gastrointestinal disorder characterized by chronic or recurrent symptoms attributed to the intestines including abdominal pain, bloating, and alternations in bowel habits. There are no obvious structural abnormalities, but the whole intestine has an excessive or abnormal response to the stimulation. Epidemiologic studies [1, 2] indicated that the prevalence of IBS in Western and Asian countries was from 15% to 24% and from 5% to 10%, respectively. Currently, though standard medical treatment such as antidepressants, antispasmodics, 5-HT3 receptor antagonists, 5-HT4 agonists, and probiotics was commonly applied for IBS, the effect is still unsatisfactory. Chinese herbal medicine has been widely used in the treatment of IBS, and some systematic reviews and meta-analysis [3–5] showed that herbal medicine had a great potential benefit in treating IBS.

According to TCM theory, treatment based on syndrome differentiation (“*bian zheng lun zhi*” in Chinese), is the basic principle of identifying and treating disease in TCM. Syndrome (*Zheng*) is a presentation of the pathological changes of a certain disease course, revealing the location, cause and nature of a disease, the correlation between pathogenic factors and healthy factors, and the body's ability to resist a disease. The aim of syndrome differentiation is based on data collected from four diagnostic methods (inspection, listening and smelling, inquiry, and palpation), symptoms, and signs to reveal the location and nature of the disease and provide the best treatment for patients [6]. Therefore, syndrome differentiation is precondition and fundamental for treatment. Since disease is a dynamic process, there may be different syndromes in different phases of a disease. So the exact syndrome differentiation can help doctors to select right herbal formula to provide the best treatment for patients in certain phases of a disease.

It is believed that individualized, syndrome differentiation-based TCM treatment would be more effective than nondifferentiation-based TCM treatment [7]. However, previous systematic reviews or meta-analysis [8, 9] published regarding the effectiveness of Chinese herbal medicine for IBS paid little attention to the application of syndrome differentiation. We searched the published or unpublished randomized controlled trials of Chinese herbal medicine for the treatment of IBS based on a Cochrane systematic review, assessed the application of syndrome differentiation among these randomized controlled trials, and analyzed the basic characteristics of studies which applied the syndrome differentiation, in order to provide the basis for further studies regarding Chinese herbal medicine based on syndrome differentiation for IBS.

## 2. Materials and Methods

### 2.1. Source of the Literature and Search Strategy

The following electronic databases were searched, regardless of publication status: the Chinese National Knowledge Infrastructure Database (CNKI) (1979–2012), the Chinese Science and Technology Periodical Database (VIP) (1989–2012), the Chinese Biomedical Database (CBM) (1978–2012), the Wanfang Database (1985–2011), PubMed (1966–2012), and the Cochrane Library (Issue 1, 2012). All the searches ended in March 2012. The search terms included “*chang_yi_ji_zong_he_zheng*” (irritable bowel syndrome), “*zhong_yi*” (Chinese medicine), “*zhong_yao*” (Chinese herbs), “*zhong_yi_yao*” (traditional Chinese medicine), “*zhong_cheng_yao*” (Chinese patent medicine), “*zhong_cao_yao*” (Chinese herb medicine), “*zhong_xi_yi_jie_he*” (integrated traditional and Western medicine), “*sui_ji*” (randomized), “*dui_zhao*” (controlled), “irritable bowel syndrome,” “herb,” “Chinese medicine,” “traditional medicine,” “plant extracts,” “alternative medicine,” “complementary medicine,” “randomized controlled trial,” and “controlled clinical trial.”

### 2.2. Inclusion Criteria

Studies meeting the following four criteria were included. (1) Randomized controlled trials, regardless of publication status; (2) participants with IBS, regardless of age, sex, ethnic origin, and clinical type (diarrhea or constipation predominated); (3) Chinese herbal medicine, regardless of formulation, preparation, dosage, or delivery. The control intervention could be no treatment, placebo, or conventional medicine. Co-intervention was also allowed if they were applied in all arms; (4) clinical outcomes included symptoms, quality of life, recurrence, number, and type of adverse events.

### 2.3. Exclusion Criteria

The following studies were excluded. (1) Duplication: the same data of patients with the same authors published in different journals; (2) information of participants, interventions, or outcomes were not available.

### 2.4. Study Selection and Data Extraction

Two authors (Q. Li and G.-Y. Yang) selected studies independently according to the inclusion and exclusion criteria. We used Epidata 3.1 software to develop a predesigned data extraction form, and two authors (Q. Li and G.-Y. Yang) extracted data independently. The following data were extracted: study design and setting, clinical type of IBS, diagnostic criteria, criteria of syndrome differentiation, inclusion and exclusion criteria, herbal medicine (name of herbs, preparation, means of delivery, and duration of intervention), control, outcome measures, and number and type of adverse events.

### 2.5. Risk of Bias Assessment

The following items were independently assessed by two authors (Q. Li and G.-Y. Yang) using the Cochrane risk of bias tool recommended by the Cochrane Reviewer's Handbook 5.0.2 [10]: random sequence generation, allocation concealment, blinding, completeness of outcome data, selective outcome reporting, and other potential source of bias. The risk of bias for each item was graded as “low risk,” “unclear risk,” or “high risk.” Disagreements were submitted to J.-P. Liu to resolve.

### 2.6. Data Analysis

Data were presented as frequency or percentage. *t*-test or Wilcoxon rank sum test was used for the comparisons of quantitative data between groups, while *χ* ^2^ test was used for the comparisons of qualitative data between groups. SPSS 17.0 software was used for all the data analysis. *P* < 0.05 was considered to be statistically significant.

## 3. Results

### 3.1. Literature Research and Study Selection

**Figure 1:**
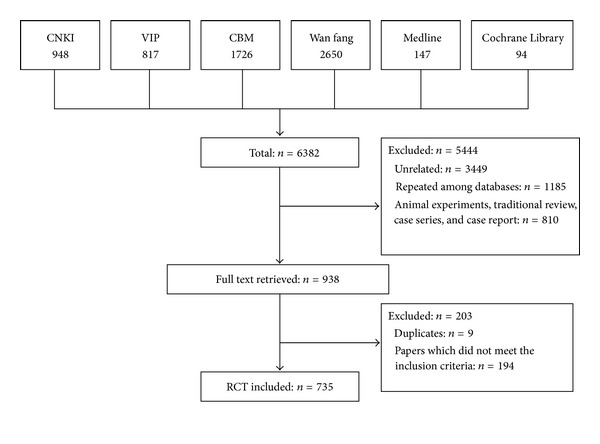
Flowchart of the literature search and study selection.

Our initial searches identified 6,382 references: 6,141 from Chinese databases and 241 from English databases. After study selection, a total of 735 randomized controlled trials were included (Figure 1).

### 3.2. Description of Included Studies

 In total, 735 RCTs involving 67,784 IBS patients were included in this paper. The average sample size was 92 patients (ranging from 20 to 360). The majority of studies were carried out in China (724/735, 98.5%). 383 (383/735, 52.1%) studies enrolled IBS patients with diarrhea-predominant type, 80 (80/735, 10.9%) studies enrolled those with constipation-predominant type, 6 (6/735, 0.8%) studies enrolled both types of IBS, and 266 (266/735, 36.2%) studies did not specify the type of IBS in their patients. 488 (488/735, 66.4%) studies used Rome criteria or Manning criteria for the diagnosis of IBS, 174 (174/735, 23.7%) studies used Chinese national criteria, 24 (24/735, 3.3%) studies used self-defined diagnostic criteria mainly based on Rome criteria, and the remaining 49 (49/735, 6.7%) studies did not specify the diagnostic criteria. The majority of studies (584/735, 80.8%) used herbal decoction. The control intervention was placebo in 21 (21/735, 2.9%) studies, no intervention in one (1/735, 0.1%) study, and conventional medicine in 534 (534/735, 72.7%) studies. 179 (179/735, 24.4%) studies compared herbal medicine combined with conventional medicine versus conventional medicine alone. All studies reported outcome of the IBS related symptoms, 24 (24/735, 3.3%) studies reported quality of life, 97 (97/735, 13.2%) studies reported relapse, and 193 (193/735, 26.3%) studies reported outcome of adverse events.

### 3.3. Risk of Bias in Included Studies

Of the 735 included studies, 79 (79/735, 10.7%) specified the methods for generation of random sequence. Among them, 53 (53/79, 67.1%) studies used random number table or computer-generated numbers, 17 (17/79, 21.5%) studies used statistical software such as SAS or SPSS, eight (8/79, 10.1%) studies used drawing lots, and one (1/79, 1.3%) study used coin tossing. Ten (10/735, 1.4%) studies provided information about allocation concealment. Among them, eight (8/10, 80.0%) studies used sealed envelope, and two (2/10, 20.0%) studies used central control for the allocated treatment. Blindings were reported in 25 (25/735, 3.4%) studies. Among them, 14 (14/25, 56.0%) studies reported double blinding, and 11 (11/25, 44.0%) studies reported single blinding. 25 (25/735, 3.4%) studies reported the information of withdrawal and dropout, and 12 (12/25, 48.0%) studies applied intention-to-treat analysis. 66 (66/735, 9.0%) studies were judged with high risk as some prespecified outcomes were not reported in the results. 11 (11/735, 1.5%) studies reported a pretrial estimation of sample size.

In conclusion, the general methodological quality of most included trials was unclear or high risk of bias.

### 3.4. Application Condition of Syndrome Differentiation in Included Studies

Of the 735 studies, 224 (224/735, 30.5%) applied syndrome differentiation and reported the related data. Although 11 (11/735, 1.5%) studies mentioned syndrome differentiation in the diagnostic criteria of IBS, they did not report the results of syndrome differentiation. Therefore, in this study, we mainly analyzed the 224 studies that both applied syndrome differentiations and reported the related data.

Among the 224 studies, 153 (153/224, 68.3%) studies specified the diagnostic criteria for TCM syndrome differentiation, and following was the most commonly used criteria: Clinical Research Guidelines for New Chinese Herbal Drug [11], Chinese Internal Medicine, and the Diagnosis and Treatment Protocol of Integrated Chinese and Western Medicine for Irritable Bowel Syndrome (draft) [12]. There were two types of syndrome differentiation, 143 (143/224, 63.8%) studies conducted syndrome differentiation prior to inclusion of participants, and a specified TCM syndrome was one of their inclusion criteria. These studies only enrolled patients with a specified TCM syndrome. The remaining 81 (81/224, 36.2%) studies conducted syndrome differentiation after inclusion of participants, and their inclusion criteria did not involve certain TCM syndrome(s). In addition, the majority of studies (166/224, 74.1%) performed syndrome differentiation for all patients in both groups, while the other 58 studies (58/224, 25.9%) performed it only for patients in herbal group. 202 studies (202/224, 90.2%) selected herbal medicine based on relevant results of syndrome differentiation, while the other 22 (22/224, 9.8%) studies though applied syndrome differentiation, did not select herbal medicine based on the syndrome differentiation.

### 3.5. TCM Syndrome Distribution in Patients with IBS

Of the 224 studies, 143 (143/224, 63.8%) studies enrolling patients with a specific TCM syndrome were excluded because they might fail to represent an authentic overview of syndrome distribution among IBS patients. The remaining 81 (81/224, 36.2%) studies were included for analysis of TCM syndrome distribution.

A total of 48 TCM syndromes were identified from the 81 studies. The most commonly reported syndromes were the syndrome of liver stagnation and spleen deficiency (46/81, 56.8%), spleen-stomach weakness (40/81, 49.4%), spleen-kidney yang deficiency (39/81, 48.1%), and cold and heat in complexity (14/81, 29.6%). 40 of 81 studies (40/81, 49.4%) enrolled patients with diarrhea-predominant IBS, three studies enrolled patients with constipation-predominant IBS, one study enrolled patients with mixture of both types, and the remaining studies did not specify the type of IBS. Considering the few papers reporting constipation-predominant IBS and the mixture of both types of IBS, the current paper only analyzed the data of syndrome distribution in IBS patients with diarrhea-predominant type. For IBS patients with diarrhea-predominant type, the most commonly seen syndrome was spleen-stomach weakness (23/40, 57.5%), followed by spleen-kidney yang deficiency (21/40, 52.5%), liver stagnation and spleen deficiency (21/40, 52.5%), cold and heat in complexity (11/40, 27.5%), and spleen qi deficiency (9/40, 22.5%).

### 3.6. Medication Characteristics of 224 Studies Applying Syndrome Differentiation

For preparation, 190 (190/224, 84.8%) studies used herbal decoction, of which 77 studies used standardized Chinese herbal formula, while the other 113 studies used modified formula according to different syndromes. Compared with studies that enrolled patients with different TCM syndromes, we found that studies that enrolled patients with a specific TCM syndrome more frequently used Chinese patent medicine. We also found that studies that enrolled patients with different TCM syndromes were more likely to use modified herbal formula (Table 1).

### 3.7. Application of Syndrome Differentiation in Therapeutic Effect Evaluation

Among the 81 studies enrolling patients with different TCM syndromes, 15 (15/81, 18.5%) studies considered the difference of syndrome in therapeutic effect evaluation. Among these 15 studies, 13 (13/15, 86.7%) compared effect among different TCM syndromes in herbal group, of which 6 (6/13, 46.2%) studies showed that there were significant differences among different syndromes. 2 (2/15, 13.3%) studies not only compared effect among different TCM syndromes within each group, but also conducted analysis among groups and found that there were no significant differences between groups for each syndrome (Table 2).

## 4. Discussion

In TCM clinical practice, practitioners prescribed herbal formula according to syndrome type identified through inspection, smelling, requesting, and pulse taking. Applying TCM syndrome differentiation in the clinical trial may reflect the practice and inform the prescription of the treatment. Therefore, we believe that randomized trials of Chinese herbal medicine should be able to embody the special feature of TCM syndrome differentiation under the premise of ensuring high methodological quality. Among 735 randomized trials included in this paper, only a small proportion of studies applied syndrome differentiation and selected herbal formula based on syndrome differentiation. The majority of studies failed to apply syndrome differentiation, which was diverse from TCM theory and practice. We recommend two ways to conduct syndrome differentiation in TCM clinical studies: first, apply syndrome differentiation in recruitment by using certain syndrome as one of the inclusion criteria to limit subgroup populations; second, apply syndrome differentiation after the recruitment, and in therapeutic effect evaluation, make subgroup analyses based on different syndromes. If individualized herbal treatment is used, then modified herbal formula should be prescribed based on different syndromes.

In this paper, we found that the most common syndromes of IBS patients were the syndrome of liver stagnation with spleen deficiency, weakness of spleen and stomach, deficiency of spleen and kidney yang, and cold and heat in complexity. In patients with diarrhea-predominant IBS, the syndromes of weakness of spleen and stomach, deficiency of spleen and kidney yang, liver depression with spleen deficiency, cold and heat in complexity, and spleen qi deficiency are the more prevalent ones, which was consistent with the results of previous studies [13–15].

Chinese patent medicine was more frequently used in studies enrolling patients with specified syndrome, while modified decoction according to syndromes was more frequently used in studies enrolling IBS patients regardless of syndrome type. Chinese patent medicine is made from Chinese materia medica according to standardized TCM prescription, with fixed components and proportion of drugs dose. Therefore, it is different from decoction which is so flexible that can be modified based on different syndromes, and its application is only for patients with a specified TCM syndrome. It explains why Chinese patent medicine is used more frequently in studies enrolling IBS patients with a specified syndrome. 

Syndrome differentiation is the most distinctive character of TCM practice. It is believed that TCM treatment based on syndrome differentiation would be more effective; however, few studies demonstrate additional benefit from TCM syndrome differentiation. Among 735 studies included in this paper, only one trial [16] published in English compared the therapeutic effect of individualized treatment based on syndrome differentiation with that of standard Chinese herbal prescription and found that the therapeutic effect of individualized treatment based on syndrome differentiation was more sustainable in treating IBS. This study was published in 1998, and since then no other studies were published to verify this finding. Therefore, more large well-designed studies using syndrome differentiation are warranted to confirm if any additional therapeutic benefits can be achieved by using syndrome differentiation.

Although TCM syndrome differentiation was applied in some randomized controlled trials of Chinese herbal medicine for IBS, we found there were some problems in application.

1. The criteria of TCM syndrome differentiation were not clear or consistent. Among 224 studies which applied syndrome differentiation, 71 studies (31.6%) did not specify the criteria for syndrome differentiation. Since syndrome differentiation is an important part of diagnosis and treatment for patients, it is essential to use reliable and practical criteria to ensure the accuracy, consistency, and repeatability of syndrome differentiation.
2. Chinese herbal formula was not prescribed based on the syndrome differentiation. Although 22 of 224 studies applied syndrome differentiation in recruitment stage, in treatment stage patients with different syndromes were given the same herbal formula, which violated the basic principle of TCM syndrome differentiation and might affect the therapeutic effect of herbal medicine. Practitioners should make prescriptions corresponding to syndromes of patients, no matter using standard prescription or modified herbal formula. It is believed that only selecting herbal formula based on TCM syndrome differentiation could ensure clinical efficacy.
3. The methods for effect analysis were inadequate. In this paper, we found no studies used stratified randomization, and only 15 studies considered the influence of syndrome on effect in statistical analysis, of which 2 used stratification analysis, 4 analyzed the difference of effect among patients with different syndromes that received the same prescription, and 9 analyzed the difference of effect among patients with different syndromes that received different prescriptions. The majority of studies neglected syndromes' influence on effect resulting in unreliable findings and limited the value of clinical application. Since the reaction of patients with different syndromes to the same prescription is different, outcomes will also be different. Considering the influence of syndromes on effect, during recruitment stage of a randomized controlled trial, patients should be stratified according to different syndromes before randomization, or during statistical analysis stage, and perform stratification analysis to ensure objective evaluation of effect.
4. Syndrome differentiation was just performed in treatment group and not in control group. Among 224 studies using syndrome differentiation, a large number of the studies did not perform syndrome differentiation in control group. Only when syndrome differentiation is performed in both groups, the baseline information can be available about syndrome differentiation of two groups, which is very important for stratified randomization or data analysis to control the influence of syndrome on effect.

To sum up, the application of syndrome differentiation in the treatment of irritable bowel syndrome is poor. In most of the studies, the selection of Chinese prescription is not based on syndrome differentiation, which might affect the pertinence of treatment of Chinese herbs. What is worse there are a series of problems in application of syndrome differentiation. More multicentered large-scale randomized controlled trials performing appropriate TCM syndrome differentiation are warranted. We make the following recommendations for further studies. (1) Stratified randomization. All participants should be stratified according to their syndromes before randomization. It is also suggested to use a specific syndrome as one of the inclusion criteria, and only enroll patients with that specific syndrome. (2) Perform syndrome differentiation for all participants, regardless of the intervention they received. Specially, definite criteria of TCM syndrome differentiation should be reported. (3) Prescribe herbal treatment based on syndrome differentiation. (4) Report disease-related outcomes, such as endpoint, quality of life, or global improvement of symptoms through validated instrument and adverse events. Composite outcomes such as total effective rate should be avoided. (5) Use stratified analysis according to syndrome in therapeutic effect evaluation if there are different syndromes.

## Supplementary Material

Search terms and Search Strategies for individual databases.

## Figures and Tables

**Table 1:** Comparison of medication characteristics between two types of syndrome differentiation in randomized trials.

Item	Studies that enrolled patients with a specific TCM syndrome only (*n* = 143)	Studies that enrolled patients with different TCM syndromes (*n* = 81)	Statistics	*P* value
Preparation*				
Decoction	113 (79.0%)	77 (95.1%)	—	0.001
Chinese patent medicine	12 (8.4%)	0 (0.0%)		
Hospital preparation	18 (12.6%)	4 (4.9%)		
Modified				
Yes	52 (46.0%)	61 (79.2%)	*χ* ^2^ = 20.946	<0.001
No	61 (54.0%)	16 (20.8%)		
Route of delivery				
Oral	139 (97.2%)	74 (91.4%)	*χ* ^2^ = 2.635	0.105
Other	4 (2.8%)	7 (8.6%)		
Course of treatment (days)	31 ± 14	30 ± 10	*Z* = −1.645	0.100
Time of followup (days)	121 ± 83	150 ± 53	*Z* = −0.176	0.860

*Fisher's exact test was used in statistical analysis.

**Table 2:** Presentations of therapeutic effect of herbal medicine in relation to TCM syndromes among 15 RCTs.

Study ID	TCM syndrome	Herbal formula	Control	Main findings
Cheng WJ 2000*	Qi stagnation and blood stasis Spleen and stomach weakness Spleen-kidney yang deficiency Other syndromes	Modified Li Zhong Tang	Sodium cromoglycate, diazepam, vitamin B1 plus loperamide hydrochloride if diarrhea exists	Herbal medicine showed a statistically significant benefit for short-term and long-term effects compared with conventional medicine No significant difference among patients with different TCM syndromes for short-term and long-term effects in herbal group

Hu ZL 2000*	Liver stagnation and spleen deficiency Liver-stomach disharmony Spleen-stomach deficiency-cold	Jianwei Yuyang Pill	Oryzanol bifidobacterium triple viable capsules	Herbal medicine showed a statistically significant benefit for short-term and long-term effects compared with conventional Western medicine therapy In herbal group, there was no significant difference between patients with syndrome of liver depression and spleen insufficiency and syndrome of liver-stomach disharmony for short-term effect, but they all were significantly better than patients with syndrome of spleen-stomach deficiency-cold

Duan GY 2002*	Liver-stomach disharmony Spleen deficiency with excessive damp Spleen-kidney yang deficiency Yin-deficiency and congealing cold	Modified Xiao Yao San and Si Jun Zi Tang used orally; Lian Qin Tang used by retention enema Xiangsha Weiling Tang plus Pu Huang used orally; Lian Qin Tang used by retention enema Li Zhong Tang and Si Shen Wan plus Yuan Hu and Fu Pian used orally Lian Qin Tang used by retention enema Wen Pi Tang plus Dang Gui, Chuan Xiong, and Shu Di Huang used orally Lian Qin Tang used by retention enema	Oryzanol and bifidobacterium viable capsule	The total effective rate in herbal group was significantly higher than in control group In herbal group, the total effective rate in patients with syndrome of liver-spleen disharmony was the best, followed by patients with syndrome of spleen insufficiency with damp harassment, spleen-kidney yang deficiency, yin-deficiency and pathogenic cold

Tian JY 2002*	Liver qi attacking the spleen Weakness of spleen and stomach Spleen-kidney yang deficiency	Modified Si Ni San plus Tong Xie Yao Fang Modified Shenling Baizhu San Li Zhong Wan plus Si Shen Wan	Western medicine based on symptoms	Herbal medicine showed a statistically significant benefit for clinical effect compared with western medicine based on symptoms There was no significant difference among patients with different TCM syndromes for clinical effect in herbal group

Liang X 2005*	Liver stagnation and spleen deficiency Weakness of spleen and stomach Spleen-kidney yang deficiency	Modified Tong Xie Yao Fang no. 1 combined with Shi Yi Fang Modified Tong Xie Yao Fang No. 2 combined with Shi Yi Fang Modified Tong Xie Yao Fang No. 3 combined with Shi Yi Fang	Pinaverium bromide plus fluoxetine if psychiatric symptoms existed, plus alprazolam if sleep disorders existed	Herbal medicine showed a statistically significant benefit for clinical effect compared with conventional western medicine therapy In herbal group, clinical effect on patients with syndrome of liver depression with spleen insufficiency was the best, followed by patients with syndrome of weakness of spleen and stomach, and syndrome of spleen-kidney yang deficiency

Li QH 2006*	Liver stagnation and spleen deficiency Weakness of spleen and stomach Spleen-kidney yang deficiency	Modified Tong Xie Yao Fang no. 1 Modified Tong Xie Yao Fang no. 2 Modified Tong Xie Yao Fang no. 3	Pinaverium bromide plus belladonna if abdominal pain exists, plus compound diphenoxylate if diarrhea exists, plus doxepin if psychiatric symptoms existed	Herbal medicine showed a statistically significant benefit for clinical effect compared with conventional Western medicine therapy In herbal group, there was no significant difference between patients with syndrome of liver depression and spleen insufficiency and syndrome of weakness of spleen and stomach, but they all were significantly better than patients with syndrome of spleen-kidney yang deficiency

Liu AQ 2006*	Spleen-stomach deficiency-cold Cold-damp stagnation in large intestine Stagnant of qi movement	Modified Xiangsha Liujunzi Tang Modified Wei Ling Tang Modified Liu Mo Tang	Western medicine based on symptoms	Stratified analysis according to syndrome differentiation The total effective rate in herbal group was significantly higher than control group for patients with syndrome of spleen-stomach deficiency-cold The total effective rate in herbal group was significantly higher than control group for patients with syndrome of cold-damp stagnation in intestines The total effective rate in herbal group was significantly higher than in control group for patients with syndrome of stagnant of qi movement

Liang W 2007*	Liver stagnation and spleen deficiency Cold and heat in complexity Weakness of spleen and stomach	Modified Tong Xie Yao Fang no. 1 Modified Tong Xie Yao Fang no. 2 Modified Tong Xie Yao Fang no. 3	Loperamide hydrochloride	The total effective rate in herbal group was significantly higher than in control group There was no significant difference among patients with different TCM syndromes on the total effective rate in herbal group

Huang PR 2008*	Liver stagnation and spleen deficiency Cold and heat in complexity Weakness of spleen and stomach	Modified Tong Xie Yao Fang no. 1 Modified Tong Xie Yao Fang no. 2 Modified Tong Xie Yao Fang no. 3	Loperamide hydrochloride	The total effective rate in herbal group was significantly higher than in control group There was no significant difference among patients with different TCM syndromes on the total effective rate in herbal group

Leng QN 2009*	Liver stagnation and spleen deficiency Cold and heat in complexity Weakness of spleen and stomach Food stagnation in stomach and intestines Spleen-kidney yang deficiency	Modified self-prepared herbal formula no.1 Modified self-prepared herbal formula no. 2 Modified self-prepared herbal formula no. 3 Modified self-prepared herbal formula no. 4 Modified self-prepared herbal formula no. 5	Trimebutine	The total effective rate in herbal group was significantly higher than in control group There was no significant difference among patients with different TCM syndromes on the total effective rate in herbal group

Zhang YF 2009*	Liver-spleen disharmony Spleen-kidney yang deficiency Spleen qi deficiency	Modified Hu Su Gan San plus Tong Xie Yao Fang Modified Si Shen Wan Modified Shenling Baizhu San	Pinaverium bromide	The total effective rate in herbal group was significantly higher than in control group There was no significant difference among patients with different TCM syndromes on the total effective rate in herbal group.

He ZM 2010*	Liver stagnation with spleen insufficiency Cold and heat in complexity Yin deficiency and intestines dryness Intestinal constraint	Modified self-prepared Yiji Changkang Tang	Western medicine based on symptoms	Herbal medicine showed a statistically significant benefit for clinical effect compared with conventional western medicine therapy There was no significant difference among patients with different TCM syndromes for clinical effect in herbal group

Li HJ 2010**	Liver-spleen disharmony Spleen-kidney yang deficiency Cold and heat in complexity	Modified Chaihu Shugan San plus Tong Xie Yao Fang Modified Si Shen Wan plus Li Zhong Tang Modified Wu Mei Wan	Western medicine based on symptoms	The cure rate in herbal group was significantly higher than control group In herbal group, the cure rate in patients with syndrome of spleen-kidney yang deficiency was significantly higher than patients with liver-spleen disharmony and cold and heat in complexity, but there was no significant difference between patients with syndrome of liver-spleen disharmony and patients with cold and heat in complexity

Bian LQ 2011***	Liver-spleen disharmony Weakness of spleen and stomach Spleen-kidney yang deficiency	Changan Yihao Fang	Placebo	Applied stratified analysis according to syndrome differentiation The score of IBS-SSS in herbal group was significantly lower than control group There was no significant difference between two groups for patients with each syndromes

Wang C 2012*	Liver qi attacking the spleen Weakness of spleen and stomach Kidney yang deficiency	Tong Xie Yao Fang combined with trimebutine	Trimebutine	Herbal medicine showed a statistically significant benefit for clinical effect compared with trimebutine In herbal group, patients with syndrome of liver qi attacking the spleen showed the best clinical effect, followed by patients with syndrome of weakness of spleen and stomach, and patients with syndrome of kidney yang deficiency

*represents studies which are taking total effective rate as the primary outcome; **represents studies which are taking cure rate as the primary outcome; ***represents studies which are taking score of IBS-SSS as the primary outcome.
