# Supplementary material for: Syndrome Differentiation in Chinese Herbal Medicine for Irritable Bowel Syndrome: A Literature Review of Randomized Trials
Source: Evid Based Complement Alternat Med. 2013 Mar 11;2013:232147. doi: 10.1155/2013/232147 (PMC3608279; doi:10.1155/2013/232147)
Supplement: Supplementary file 1 — Search terms and Search Strategies for individual databases. [file 232147.f1.pdf]

**Appendix：Search terms and Search Strategies for individual databases**

**Search terms:**

Chinese terms: "*chang_yi_ji_zong_he_zheng*" (irritable bowel syndrome), "*zhong_yi*" (Chinese medicine), "*zhong_yao*" (Chinese herbs), "*zhong_yi_yao*" (traditional Chinese medicine), "*zhong_cheng_yao*" (Chinese patent medicine), "*zhong_cao_yao*" (Chinese herb medicine), "*zhong_xi_yi_jie_he*" (integrated traditional and western medicine), "*sui_ji*" (randomized), "*dui_zhao*" (controlled)

English terms: "irritable bowel syndrome", "herb", "Chinese medicine", "traditional medicine", "plant extracts", "alternative medicine", "complementary medicine", "randomized controlled trial", "controlled clinical trial"

**Search strategy:**

**CNKI**

1 "*chang_yi_ji_zong_he_zheng*" (Title)

2 "*zhong_yi*" (Title/Abstract)

3 "*zhong_yao*" (Title/Abstract)

4 "*zhong_yi_yao*" (Title/Abstract)

5 "*zhong_cheng_yao*" (Title/Abstract)

6 "*zhong_cao_yao*" (Title/Abstract)

7 "*zhong_xi_yi_jie_he*" (Title/Abstract)

8 "*sui_ji*" (All fields)

9 "*dui_zhao*" (All fields)

10 or/ 2-7

11 or/ 8-9

12 10 and 11

13 1 and 12

**VIP**

1 "*chang_yi_ji_zong_he_zheng*" (Title)

2 "*zhong_yi*" (Title/Abstract)

3 “*zhong_yao*” (Title/Abstract)

4 “*zhong_yi_yao*” (Title/Abstract)

5 “*zhong_cheng_yao*” (Title/Abstract)

6 “*zhong_cao_yao*” (Title/Abstract)

7 “*zhong_xi_yi_jie_he*” (Title/Abstract)

8 “*sui_ji*” (All fields)

9 “*dui_zhao*” (All fields)

10 or/ 2-7

11 or/ 8-9

12 10 and 11

13 1 and 12

**CBM**

1 “*chang_yi_ji_zong_he_zheng*” (Title)

2 “*zhong_yi*” (Default)

3 “*zhong_yao*” (Default)

4 “*zhong_yi_yao*” (Default)

5 “*zhong_cheng_yao*” (Default)

6 “*zhong_cao_yao*” (Default)

7 “*zhong_xi_yi_jie_he*” (Default)

8 “*sui_ji*” (All fields)

9 “*dui_zhao*” (All fields)

10 or/ 2-7

11 or/ 8-9

12 10 and 11

13 1 and 12

**Wanfang**

1 “*chang_yi_ji_zong_he_zheng*” (Title)

2 “*zhong_yi*” (Keyword/Abstract)

3 “*zhong_yao*” (Keyword/Abstract)

4 “*zhong_yi_yao*” (Keyword/Abstract)

5 “*zhong_cheng_yao*” (Keyword/Abstract)

6 “*zhong_cao_yao*” (Keyword/Abstract)

7 “*zhong_xi_yi_jie_he*” (Keyword/Abstract)

8 “*sui_ji*” (All fields)

9 “*dui_zhao*” (All fields)

10 or/ 2-7

11 or/ 8-9

12 10 and 11

13 1 and 12

**PubMed**

1 irritable bowel syndrome.mh

2 herb.tw

3 Chinese medicine＄.tw

4 traditional medicine＄.tw

5 plant extract＄.tw

6 alternative medicine＄.tw

7 complementary medicine＄.tw

8 randomized controlled trial.pt

9 controlled clinical trial.pt

10 or/2-7

11 or/8-9

12 10 and 11

13 1 and 12

**Cochrane library**

1 irritable bowel syndrome.mh

2 herb.tw

3 Chinese medicine.ti,ab,sh

4 traditional medicine. ti,ab,sh

5 plant extract. ti,ab,sh

6 alternative medicine. ti,ab,sh

7 complementary medicine. ti,ab,sh

8 randomized controlled trial.pt

9 controlled clinical trial.pt

10 or/2-7

11 or/8-9

12 10 and 11

13 1 and 12
